# A Compact Detection Platform Based on Gradient Guided-Mode Resonance for Colorimetric and Fluorescence Liquid Assay Detection

**DOI:** 10.3390/s21082797

**Published:** 2021-04-15

**Authors:** Jing-Jhong Gao, Ching-Wei Chiu, Kuo-Hsing Wen, Cheng-Sheng Huang

**Affiliations:** 1 Department of Mechanical Engineering, National Yang Ming Chiao Tung University, Hsinchu 30010, Taiwan; jhong.me07g@nctu.edu.tw (J.-J.G.); jerry0851020.me08g@nctu.edu.tw (C.-W.C.); 2 Degree Program of Automation and Precision Engineering, College of Engineering, National Yang Ming Chiao Tung University, Hsinchu 30010, Taiwan; khwen.ape07g@nctu.edu.tw

**Keywords:** subwavelength grating, spectral detection, guided-mode resonance, fluorescence detection, colorimetric assays, albumin, creatinine

## Abstract

This paper presents a compact spectral detection system for common fluorescent and colorimetric assays. This system includes a gradient grating period guided-mode resonance (GGP-GMR) filter and charge-coupled device. In its current form, the GGP-GMR filter, which has a size of less than 2.5 mm, can achieve a spectral detection range of 500–700 nm. Through the direct measurement of the fluorescence emission, the proposed system was demonstrated to detect both the peak wavelength and its corresponding intensity. One fluorescent assay (albumin) and two colorimetric assays (albumin and creatinine) were performed to demonstrate the practical application of the proposed system for quantifying common liquid assays. The results of our system exhibited suitable agreement with those of a commercial spectrometer in terms of the assay sensitivity and limit of detection (LOD). With the proposed system, the fluorescent albumin, colorimetric albumin, and colorimetric creatinine assays achieved LODs of 40.99 and 398 and 25.49 mg/L, respectively. For a wide selection of biomolecules in point-of-care applications, the spectral detection range achieved by the GGP-GMR filter can be further extended and the simple and compact optical path configuration can be integrated with a lab-on-a-chip system.

## 1. Introduction

Colorimetric and fluorescent assays are among the most popular techniques for quantifying target analytes in a liquid sample. Such quantification has varied applications, including food safety, water quality monitoring, biomarker detection, and pathogen detection [1,2,3,4,5]. Fluorescence detection of chemically tagged biomolecules is a widely used method in diagnostic assays, including deoxyribonucleic acid (DNA) or protein detection assays and immunoassays, due to its excellent sensitivity [6,7,8]. In fluorescent assays, the intensity of a specific wavelength can be monitored to be correlated with the analyte concentration. By contrast, colorimetric assays, such as the commonly used enzyme-linked immunosorbent assay for biomolecule detection [9], typically measure changes in the color or intensity of a liquid sample through the reaction of reagents with the targeted analyte to produce colored products [2,9]. The absorbance at a specific wavelength can be correlated with the analyte concentration in the liquid sample.

To measure the fluorescence or absorbance, a photodiode with an appropriate optical filter can be used depending on the fluorophores or colorimetric reagents [2,10]. However, in the absence of the spectral information, only the intensity variation can be determined. Spectrometers are often used to obtain both intensity and spectral information. However, traditional spectrometers are bulky, costly, and difficult to integrate with sensor chips to realize miniaturized systems for current point-of-care diagnostics [5]. Wan et al. [5] proposed a detection platform for fluorescence and absorption spectrum measurement that contains a linear variable filter on top of a complementary metal oxide semiconductor. This simple configuration has the potential of integrating biomolecule analysis capabilities into smartphones or other portable devices for point-of-care applications. The current paper proposes a miniaturized platform for fluorescence-based and colorimetric detection. The detection platform includes a gradient grating period guided-mode resonance (GGP-GMR) device on top of a linear charge-coupled device (CCD). The GGP-GMR filter exhibits spatially dependent resonance characteristics and converts spectral information into spatial information in the CCD, which allows intensity and wavelength information from fluorescence emission or colorimetric assays to be directly determined with the CCD without the requirement of a bulky and expensive spectrometer.

## 2. Materials and Methods

### 2.1. Sensor Design, Fabrication, and Characterization

Our detection platform is based on the concept of GGP-GMR, which is based on the guided-mode resonance (GMR) effect [11,12]. With an appropriate design in device geometry and material selection, at normal incidence, a specific wavelength of light (resonant wavelength) can be reflected, which results in minimum transmission; thus, GMR functions as a bandstop filter. The resonant wavelength can be calculated under the second-order Bragg condition [13], namely $\lambda_{R}=n_{eff}\Lambda$, where $\lambda_{R}$ is the resonant wavelength, $n_{eff}$ is the effective refractive index, and Λ is the grating period. The variable $n_{eff}$ can be considered as a weighted average of the refractive indices of the overall GMR structure, which can be calculated by referring to [14]. The resonant wavelength is highly sensitive to the grating period. By varying the grating period along a device, a linearly varying bandstop filter can be achieved, where the resonant wavelength varies along the device. Such a device is termed as a GGP-GMR device [15].

**Figure 1:**
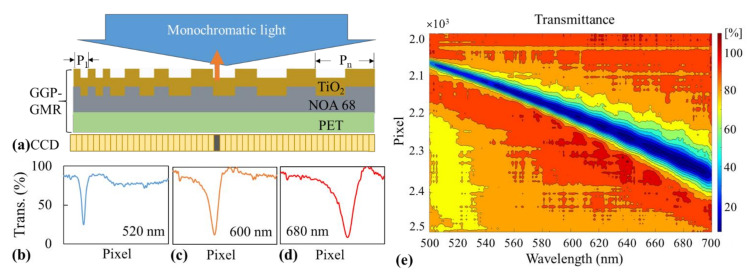
(**a**) Illustration of a three-layer gradient grating period guided-mode resonance (GGP-GMR) device on top of a charge-coupled device (CCD). The large blue arrow indicates an incident light with a specific wavelength, and the orange arrow indicates the light reflected at a specific grating period. The reflected light indicated by the orange arrow results in the minimum intensity in the underneath CCD pixel (represented in dark gray). Intensity distribution on the CCD for incident wavelengths of (**b**) 520, (**c**) 600, and (**d**) 680 nm. (**e**) Calibration diagram of the transmission efficiency at each pixel for each wavelength.

The GGP-GMR device used in this study comprises a simple three-layer structure consisting of a polyethylene terephthalate (PET) substrate, an optical adhesive (Norland 68 [NOA 68], Norland Products Inc., Cranbury, NJ, USA) replicated gradient grating structure, and a TiO_2_ waveguiding layer (Figure 1a). The detailed design and fabrication of this GGP-GMR device are highly similar to those in our previous study [15]. In brief, the GGP-GMR device used in this study has a grating period varying from 250 to 550 nm in increments of 2 nm. Each period comprises 100 repeated cycles. A GGP-GMR device with grating periods ranging from 250 to 550 nm can support resonant wavelengths with a bandwidth of over 400 nm [15]; however, for the purpose of this study, only grating periods corresponding to resonant wavelengths between 500 and 700 nm were used to demonstrate the fluorescence and colorimetric detection ability of the developed device.

To fabricate the device used in this study, first, electron beam lithography and reactive ion etching were used to pattern the gradient grating period of a Si master with a grating depth of 85 nm and a duty cycle of approximately 0.5. The liquid form of NOA 68 was then sandwiched between the Si master and a flexible PET sheet. Once NOA 68 was solidified with ultraviolet light exposure, the NOA 68/PET with the replicated gradient grating pattern was separated from the Si master. Finally, a TiO_2_ layer (~130 nm thick) was sputter-coated on top of NOA68/PET. A detailed discussion on the device geometry and optical characterization can be found in [15].

The GGP-GMR device exhibits linearly varying resonance, as illustrated in Figure 1a. For a specific monochromatic illumination, the light resonates (and reflects) in a specific period, as illustrated by the orange arrow in Figure 1a, and is transmitted in other periods, which results in the minimum intensity underneath this period, as represented by the dark gray pixel on the CCD in Figure 1a. To examine the spatially dependent resonance, a broadband-light-coupled (LSH-150, Taiwan Fiber Optics, Inc., Taipei, Taiwan) monochromator (DK242, Spectral Products, Putnam, CT, USA) was used to generate a narrowband light source with a linewidth of approximately 2.5 nm. The light was coupled with a 600-μm optical fiber and collimated before illuminating the GGP-GMR device. A combination of short- and long-pass filters was used to limit the spectral range between 500 and 700 nm.

The transmitted light was recorded using a linear CCD (LHCCD01304, ToupTek Photonics Co., Hangzhou, Zhejiang, China). Figure 1b–d presents the transmission efficiency recorded by the linear CCD at three incident wavelengths—520, 600, and 680 nm. The aforementioned figure clearly indicates that the light reflects at different locations (periods), which results in minimum intensities at different pixels corresponding to these periods. By scanning wavelengths from 500 to 700 nm with increments of 1 nm, the relationship between a specific incident wavelength and the minimum intensity (or transmission efficiency) corresponding pixel (MICP) can be obtained, as illustrated in Figure 1e and Figure S1 in Supplementary Material.

### 2.2. Detection Mechanism and Experimental Setup

As indicated in Figure 1e, as the incident wavelength varies from 500 to 700 nm, the MICP varies from the 2073th pixel to the 2361th pixel, which indicates a one-to-one correspondence between the wavelength and MICP; that is, for each incident wavelength, one corresponding MICP exists on the CCD. A transmission efficiency matrix *T* can be obtained from Figure 1e and used to represent this correlation. The variable *T_ij_* can be used to represent the elements in *T*. This variable indicates the transmission efficiency at the MICP *i* for the incident wavelength *j*. For example, *T*_5,100_ represents the transmission efficiency for the 100th wavelength at the fifth MICP. In this study, both *i* and *j* ranged from 1 to 200. An incident spectrum can be digitized and represented by a single-column matrix, where the elements are denoted by *I_j_*, which indicates the intensity of the *j*th wavelength, where *j* ranges from 1 to 200 and represents wavelengths of 500–700 nm in 1-nm increments. The intensity measured on the CCD can then be calculated using the formula *C* = *TI*, where *C* is a column vector of size 200 and each element represents the intensity at each MICP on the CCD.

**Figure 2:**
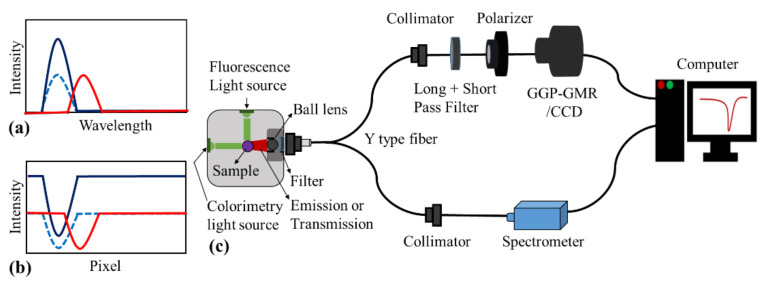
(**a**) Illustration of the incident spectrum for the GGP-GMR device, (**b**) intensity distribution on the CCD of the GGP-GMR device, and (**c**) experimental setup for the fluorescence and colorimetric assay measurement.

For the incident spectrum presented as a blue curve in Figure 2a, which is obtained for assays such as a fluorescence emission or colorimetric assay, the CCD intensity distribution illustrated as a blue curve in Figure 2b can be obtained using a simple matrix multiplication. The MICP in Figure 2b corresponds to the peak wavelength based on the calibration diagram presented in Figure 1c. When the intensity of the incident spectrum is reduced without changing its center wavelength, as indicated by the blue dashed line in Figure 2a, the corresponding intensity distribution in the CCD is also reduced, as illustrated by the blue dashed curve in Figure 2b, without changing the MICP. If both the incident wavelength and intensity are varied, as indicated by the red curve in Figure 2a, the intensity and location of the MICP change accordingly. In summary, the peak wavelength and intensity of the incident illumination are correlated with the location and intensity of the MICP in the GGP-GMR/CCD system that does not contain a bulky spectrometer.

The experimental setup presented in Figure 2c was used to demonstrate that the proposed GGP-GMR device and CCD can be used as a compact platform for fluorescence and colorimetric assay detection. A customized sample chamber was used to hold the cuvette and connect the light source and optical fiber. For fluorescence measurement, the excitation light was set perpendicular to the detection optics to minimize the interference. By contrast, for colorimetric detection, the light source was placed in line with the detection optics to measure the transmission. To evaluate the performance of the proposed GGP-GMR/CCD detection platform, the light emitted from the fluorophores or transmitted through the samples was collected by a ball lens and coupled to a 2 × 1 fiber, whose one end was connected to a commercial spectrometer (2000+VIS-NIR-ES, Ocean Inside, Orlando, FL, USA) and other end was connected to the GGP-GMR/CCD system. Depending on the assays and the excitation light source, a filter might need to be used before the light is coupled to the 2 × 1 fiber. In this study, the spectral range in our GGP-GMR/CCD system was limited between 500 and 700 nm through a combination of long- and short-pass filters illustrated in Figure 2c. The pictures of GGP-GMR on top of the CCD and the customized sample chamber can be found in Figure S2 in Supplementary Material.

## 3. Results and Discussion

### 3.1. Measurement of Fluorophore Emission

To verify that the proposed detection platform (GGP-GMR/CCD system) can detect both the intensity and peak wavelength, one fluorescent dye that is commonly used in the polymerase chain reaction for DNA detection was tested. The performance of the GGP-GMR/CCD system was compared with that of a commercial spectrometer to demonstrate its capability and flexibility for general fluorescence detection in liquid assays.

**Figure 3:**
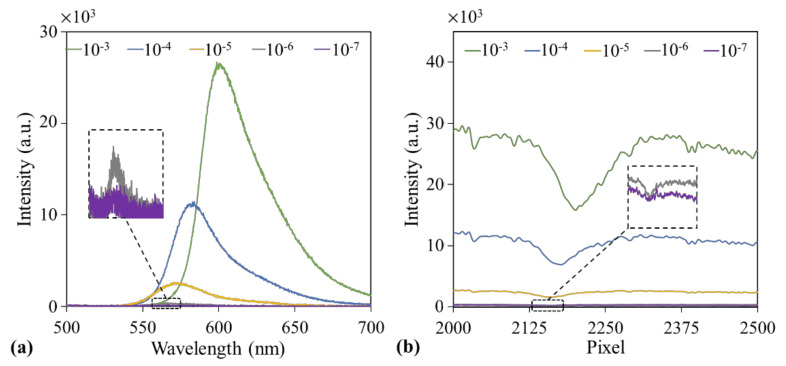
Fluorescence emission from different concentrations of TAMRA (5-carboxytetramethylrhodamine) measured using (**a**) a spectrometer and (**b**) the proposed GGP-GMR/CCD system.

Figure 3a,b presents the fluorescence intensities measured using a spectrometer and the GGP-GMR/CCD system, respectively, for five concentrations (10^−3^ to 10^−7^ M in 10-fold dilution) of TAMRA (5-carboxytetramethylrhodamine, Abcam, Cambridge, UK) in methanol. A 458-nm LED was used as the excitation source, and the fluorescence emission collected by the ball lens was coupled to the 2 × 1 fiber for the measurement (Figure 2c).

The results presented in Figure 3a indicate that the fluorescence intensity increased with increases in the analyte concentration from the spectrometer measurement, as expected. In addition, the intensity at the MICP measured by the GGP-GMR/CCD system also increased with the aforementioned concentration, as indicated in Figure 3b (explained in the previous section as well as in Figure 2a,b).

**Figure 4:**
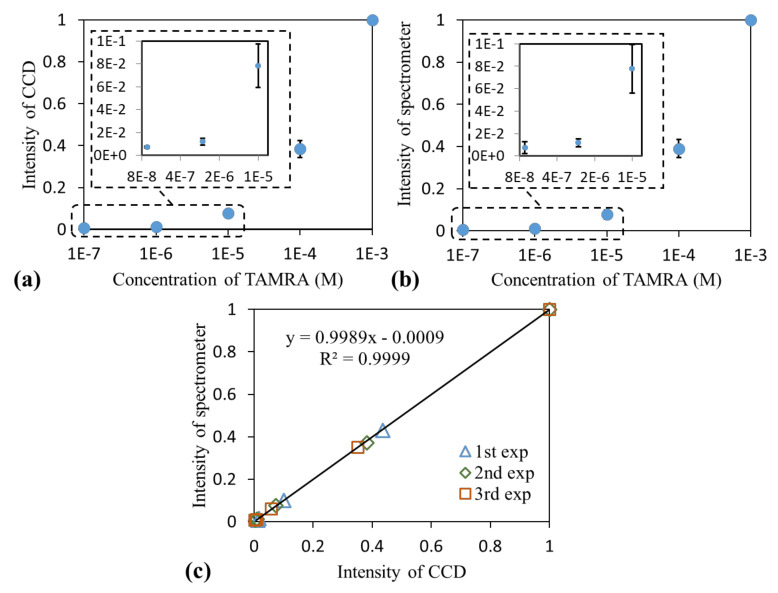
Dose response curve obtained using the (**a**) GGP-GMR/CCD system and (**b**) spectrome-193 ter. (**c**) This figure displays the correlation between the normalized intensity of the peak wave-194 length and that of the minimum intensity corresponding pixel (MICP).

Experiments were performed three times to obtain a quantitative measurement. For each run, the intensities were normalized to that measured at the highest concentration. The dose response curves measured using the GGP-GMR/CCD system and spectrometer are displayed in Figure 4a,b, respectively. A comparison of the results (normalized intensity of the peak wavelength vs. the normalized intensity of the MICP) is illustrated in Figure 4c, where both the slope of the regression line and the coefficient of determination (R2) approach 1, which indicates that the measurements obtained with the GGP-GMR/CCD system agree well with those obtained with a commercial spectrometer.

**Figure 5:**
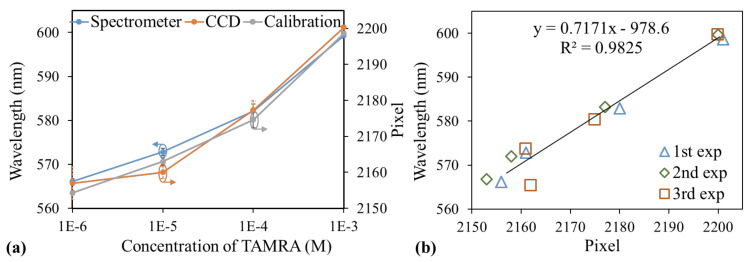
(**a**) Peak wavelength (blue), measured MICP (orange), and expected MICP (gray) based on the calibration diagram as a function of the concentration. (**b**) Correlation between the peak wavelength and the MICP.

In addition to the intensity, the peak wavelength and MICP shifted with changes in the concentration, as displayed in Figure 2a,b. The peak wavelength shifted from 600 to 566 nm when the concentration decreased from 10^−3^ to 10^−6^ M, as depicted in Figure 3a. According to the calibration diagram presented in Figure 1e, the MICP should correspond to pixel numbers of 2199–2154, which is close to the observation in Figure 3b, where the MICP corresponds to pixel numbers of 2201–2160. The variations in the peak wavelength and MICP with the concentration obtained from the three experimental runs are illustrated as blue and orange curves in Figure 5a, respectively.

Moreover, on the basis of the calibration diagram presented in Figure 1e, the calibrated MICPs are plotted as a gray curve in Figure 5a. A comparison of the results between the measured MICPs and peak wavelength measured by the spectrometer is presented in Figure 5b, where the large value of the coefficient of determination (0.9825) indicates a strong correlation between the MICP and the peak wavelength. These fluorescence measurement results indicate that the location and intensity of the MICP can be used to correlate the peak wavelength and its intensity appropriately. Similar results (Figures S3–S5) were obtained from another commonly used fluorophores, FAM (6-carboxyfluorescein, Merck, Darmstadt, Germany), which can be found in Supplementary Material.

### 3.2. Practical Assay Detection

Albumin and creatinine, which are the commonly used biomarkers for kidney diseases [16,17], were used to demonstrate that the proposed GGP-GMR/CCD system can be an effective measurement tool for practical fluorescent and colorimetric assays.

#### 3.2.1. Fluorescence Detection for Albumin Quantification

In this study, the nonimmunological method for urinary albumin determination was used to demonstrate the fluorescence detection capability of the GGP-GMR/CCD system. The detection mechanism is fairly simple. The albumin blue 580 (AB580) dye can bind to human albumin [8,18] and enhance the fluorescence quantum efficiency by two orders of magnitude [19].

The composition of artificial urine used in this study, which was obtained by referring to [20], is listed in Table S1. By spiking human serum albumin (HSA; Abcam) in the artificial urine, samples with seven HSA concentrations (200, 150, 100, 50, 25, 12.5, and 6.25 mg/L) and a blank sample (only artificial urine) were prepared. Then, 4 μL of AB580 and 46 μL of albumin assay buffer (both purchased from Abcam) were added to 50 μL of each sample with HSA. The samples were wrapped in an aluminum foil for 30 min at room temperature before the measurement.

**Figure 6:**
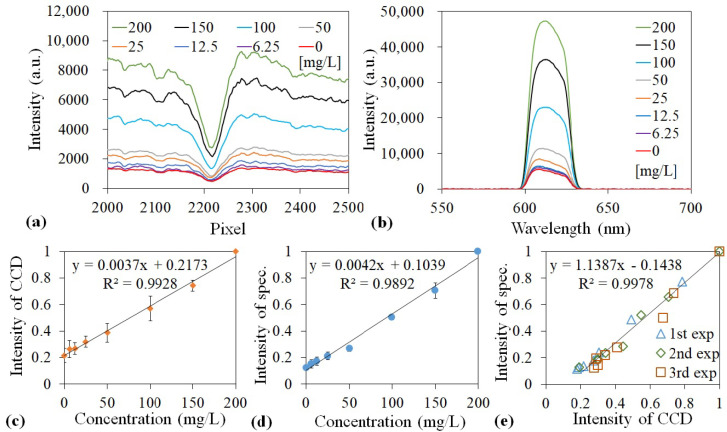
Intensity measured with the (**a**) GGP-GMR/CCD system and (**b**) spectrometer for different human serum albumin (has) concentrations. Normalized intensities for the (**c**) MICP and (**d**) peak wavelength as a function of the HSA concentration. (**e**) This figure depicts the correlation between the HSA measurements obtained from the spectrometer and GGP-GMR/CCD system.

A 5-mW green laser with a wavelength of 532 nm was used to excite the AB580-HSA conjugate. In addition, a bandpass filter with a wavelength of 605–625 nm was used to block the excitation illumination. The intensity measured by the GGP-GMR/CCD system and the emission spectra measured using a spectrometer at different concentrations are illustrated in Figure 6a,b, respectively. Both the peak wavelength intensity and MICP intensity increased with the HSA concentration. The peak wavelength fluctuated marginally between 608 and 612 nm. Moreover, the MICP fluctuated between the 2210th and 2216th pixels, which agrees well with the calibration diagram presented in Figure 1e.

The MICP and peak wavelength intensities were normalized to the highest HSA concentration (200 mg/L). The dose response curves obtained from the three experimental runs are displayed in Figure 6c,d. The correlation between the spectrometer and GPP-GMR/CCD system measurements is depicted in Figure 6e, where the slope of the regression line and the coefficient of determination (R2) approach 1, which verifies that the measurement obtained with the GGP-GMR/CCD system agrees comprehensively with that measured with a commercial spectrometer.

The limit of detection (LOD) was calculated by dividing the measurement noise with sensitivity. The noise was calculated as three times the standard deviation from all measurements. The sensitivity was obtained from the slope of the graphs presented in Figure 6c,d. The results indicate that the GGP-GMR/CCD system and spectrometer could achieve LODs of 40.99 and 17.39 mg/L, respectively, for HSA, which agreed well with the manufacturer’s manual (which states that an LOD of 20 mg/L can be achieved). Within the range of 6.25–200 mg/L, both GGP-GMR/CCD system and spectrometer exhibit a fairly linear response. The marginally better LOD achieved with the spectrometer than with the proposed device is attributable to the marginally higher sensitivity (0.042 vs. 0.037) and smaller measurement noise (0.0730 vs. 0.1517) of the spectrometer. An improved optical path design can increase the light intensity on the CCD and increase shielding on the GGP-GMR/CCD setup to prevent environmental influences. Such a design can further reduce the measured standard deviation and improve the LOD. Nevertheless, the GGP-GMR/CCD system can accurately obtain the peak wavelength and intensity information for a liquid sample.

#### 3.2.2. Colorimetric Measurement for Creatinine and Albumin Quantification

**Figure 7:**
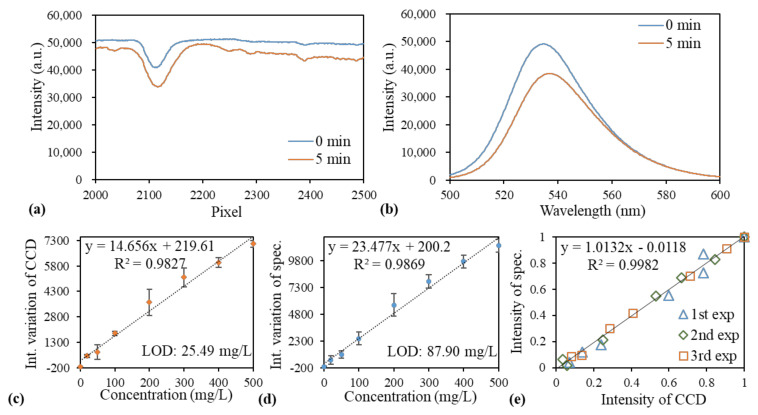
Intensity measured by the (**a**) GGP-GMR/CCD system and (**b**) spectrometer between 0 and 5 min at a creatinine concentration of 500 mg/L. The dose response curves of the normalized intensity difference between 0 and 5 min as a function of concentration measured with the (**c**) GGP-GMR/CCD device and (**d**) spectrometer. (**e**) Correlation between the two measurement systems.

The creatinine assay kit (BioChain Institute Inc., Newark, CA, USA) based on the Jaffe method [21] was used to demonstrate the quantification of the creatinine concentration. The yellow picrate solution turned red upon forming a picrate–creatinine complex in an alkaline medium. Samples with seven concentrations of creatinine (500, 400, 300, 200, 150, 100, and 50 mg/L) prepared by diluting the creatinine standard provided by the manufacturer in deionized water and one blank sample (0 mg/L creatinine, deionized water only) were used to generate the standard curve and evaluate the GGP-GMR/CCD detection system. The working reagent containing picrate, which was also provided by the manufacturer, was added to each concentration to form the complex. An LED with a wavelength of 535 nm was used to illuminate the liquid sample. The transmitted intensity and spectrum were recorded immediately after loading the working reagent (0 min) and after 5-min incubation. Figure 7a,b present the spectra recorded for the sample with 500 mg/L of creatinine by using the GGP-GMR/CCD system and spectrometer, respectively. The varying intensities between 0 and 5 min at 535 nm and the 2100th pixel for different concentrations were used to construct the dose response curves for the GGP-GMR/CCD device and spectrometer (Figure 7c,d, respectively). The intensity variation increased with the creatinine concentration, with the largest intensity variation observed at the highest concentration of 500 mg/L. The correlation between the measurement results obtained with the spectrometer and GGP-GMR/CCD device by normalizing the intensity variation at each concentration to 500 mg/L is illustrated in Figure 7e. The slope and R2 values indicate a comprehensive agreement between the results obtained with the spectrometer and proposed device.

The dose response curves displayed in Figure 7c,d indicate that the results obtained with the spectrometer and proposed system were highly close. Within the range of 50–500 mg/L, both GGP-GMR/CCD system and spectrometer exhibit a fairly linear response. The spectrometer had higher sensitivity and noise than the proposed system did, which resulted in the spectrometer having a marginally higher LOD. However, the LOD values obtained with both systems were higher the value suggested by the manufacturer, namely 1 mg/L. The main reason for this result is the continuous variation in intensity due to the persistent reaction between creatinine and the working reagent. To overcome this problem, the experiment must be executed in a highly proficient manner and the environmental lighting must be strictly controlled. Nevertheless, the location and intensity of the MICP obtained using the GGP-GMR/CCD system exhibit a suitable correlation with the peak wavelength and its intensity obtained using the spectrometer.

**Figure 8:**
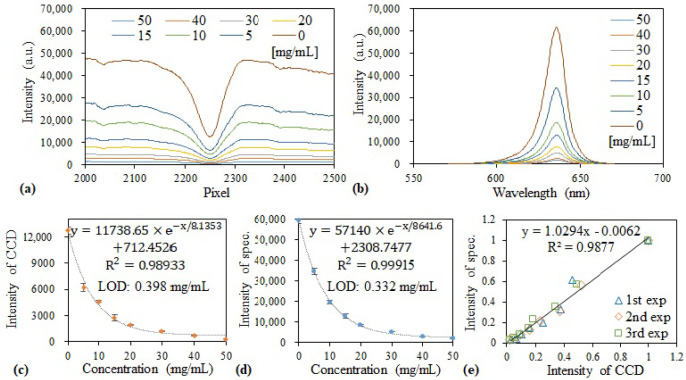
Transmission measured with the (**a**) GGP-GMR/CCD and (**b**) spectrometer. Dose response curves of the (**c**) MICP intensity and (**d**) peak wavelength intensity as functions of the albumin concentration. (**e**) Correlation between the two measurement systems.

Another example of a colorimetric assay is albumin detection based on its binding with bromocresol green. On the formation of the albumin–bromocresol green complex, the color of the assay solution changes from green to blue, and the peak absorbance is observed at 620 nm. The assay was fairly straightforward according to the manufacturer’s manual. We simply mixed different concentrations of albumin in deionized water (5–50 mg/mL) and the bromocresol green reagent provided by the manufacturer in a 1:40 ratio in a cuvette and incubated the mixtures for 5 min. An LED with a wavelength of 625 nm was used to illuminate the liquid samples. Due to the high absorbance at 620 nm upon complex formation, the transmitted intensity decreased with an increase in the albumin concentration for the measurements obtained with the GGP-GMR/CCD system and spectrometer (Figure 8a,b, respectively). The peak wavelength was approximately 636 nm. By contrast, the MICP had pixel numbers of 2247–2251, which agrees well with the calibration diagram presented in Figure 1e.

The MICP intensity and peak wavelength intensity are illustrated as functions of the concentrations obtained from the three experimental runs in Figure 8c,d, respectively. The assay did not exhibit linear responses for both systems, which agree with manufacturer’s data sheet. The LODs achieved with the proposed device and spectrometer were 0.4 and 0.33 mg/mL, respectively, which are close to the value suggested in the manufacturer’s manual (0.1 mg/mL). The correlation of the normalized intensity between the two measurement systems is depicted in Figure 8e, which indicates a good agreement between the normalized intensity results obtained with the two systems.

## 4. Conclusions

This paper proposes a GGP-GMR system with a linear CCD as a miniaturized solution for liquid assay detection based on fluorescence-based and colorimetric measurements. A comparison of the proposed system with a commercial portable spectrometer indicated that the location and intensity of the MICP obtained using the proposed system effectively correlated with the peak wavelength and its corresponding intensity obtained with the spectrometer. The results of the serial dilution tests in practical assays based on fluorescence-based and two colorimetric measurements revealed that the GGP-GMR/CCD system achieved comparable sensitivity and LOD results to those obtained of the commercial spectrometer.

For the practical fluorescent assays for AB580 and the colorimetric assay for albumin quantification, the LODs obtained with the two detection systems were very close to the value recommended by the manufacturer. By contrast, the LODs observed in the colorimetric assay for creatinine detection for the GGP-GMR/CCD system and spectrometer were worse than that suggested by the manufacturer. The main reason for this result is the relatively proficiency in performing the experiments and the marginal environmental lighting fluctuation. A better shield or package for the developed system is expected to reduce the recorded intensity fluctuation under environmental lighting to reduce the experimental noise and thus further improve the LOD. Nevertheless, the results obtained with the GGP-GMR/CCD system agreed well with those obtained using a commercial spectrometer for all three practical assays.

In summary, a GGP-GMR filter with a length of less than 2.5 mm can achieve a spectral detection range of 500–700 nm, which can be further extended to cover additional assays. Through a direct measurement of the location and intensity of the MICP on the CCD, the peak wavelength and its intensity obtained from the fluorescent or colorimetric assays can be determined without using a bulky spectrometer. The compact size of the GGP-GMR device and its simple optical path design configuration with a CCD enable accurate assay detection based on fluorescence emission and absorption measurement; thus, the proposed device has the potential to be integrated with microfluidic chips, portable devices, or smartphones for various point-of-care applications.

## Data Availability

Data available on reasonable request from the corresponding author.

## Supplementary Materials

The following are available online at https://www.mdpi.com/article/10.3390/s21082797/s1, Figure S1: The relationship between the MICP and the incident wavelength, Figure S2: (a) GGP-GMR on top of a CCD. (b) Customized sample chamber with sample inside. (c) Customized sample chamber with lid, Figure S3: Fluorescence emission from different concentrations of FAM measured using (a) a spectrometer and (b) the proposed GGP-GMR/CCD system, Figure S4: Dose response curve obtained using the (a) GGP-GMR/CCD system and (b) spectrometer. (c) This figure displays the correlation between the normalized intensity of the peak wavelength and that of the MICP, Figure S5: (a) Peak wavelength (blue) and measured MICP (orange) as a function of the concentration. (b) Correlation between the peak wavelength and the MICP, Table S1: Composition of artificial urine.
