# Supplementary material for: A Compact Detection Platform Based on Gradient Guided-Mode Resonance for Colorimetric and Fluorescence Liquid Assay Detection
Source: Sensors (Basel). 2021 Apr 15;21(8):2797. doi: 10.3390/s21082797 (PMC8071489; doi:10.3390/s21082797)
Supplement: Supplementary file 1 [file sensors-21-02797-s001.zip › sensors-1174006-supplementary.pdf]

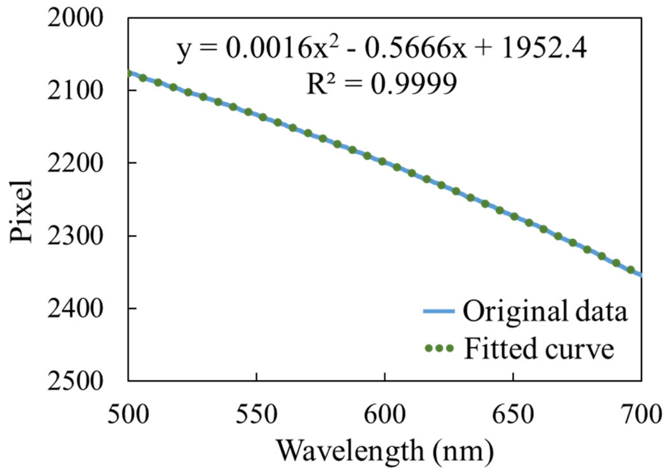

Figure 1. The relationship between the MICP and the incident wavelength.

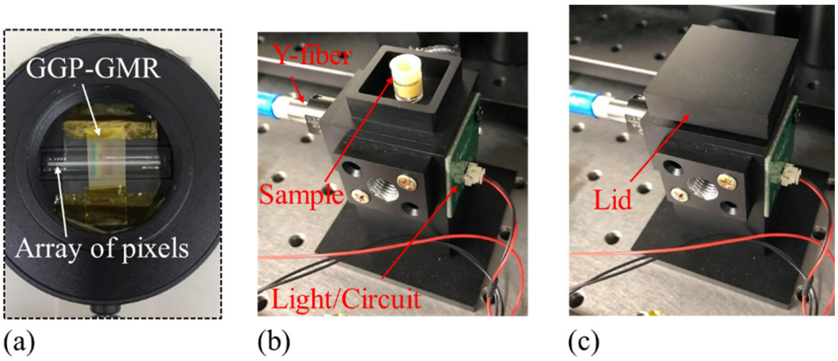

Figure S2. (**a**) GGP-GMR on top of a CCD. (**b**) Customized sample chamber with sample inside. (**c**) Customized sample chamber with lid.

Measurement of fluorescence emission of FAM

Figures S3a and S3b presents the fluorescence intensities measured using a spectrometer and the GGP-GMR/CCD system, respectively, for five concentrations (10^{-3} to 10^{-7} M in 10-fold dilution) of FAM (6-carboxyfluorescein, Merck) in 0.1 N NaOH. A 458-nm LED was used as the excitation source, and the fluorescence emission collected by the ball lens was coupled to the 2 × 1 fiber for the measurement.

The results presented in Figure S3a indicate that the fluorescence intensity increased with increases in the analyte concentration from the spectrometer measurement, as expected. In addition, the intensity at the MICP measured by the GGP-GMR/CCD system also increased with the aforementioned concentration, as indicated in Figure S3b.

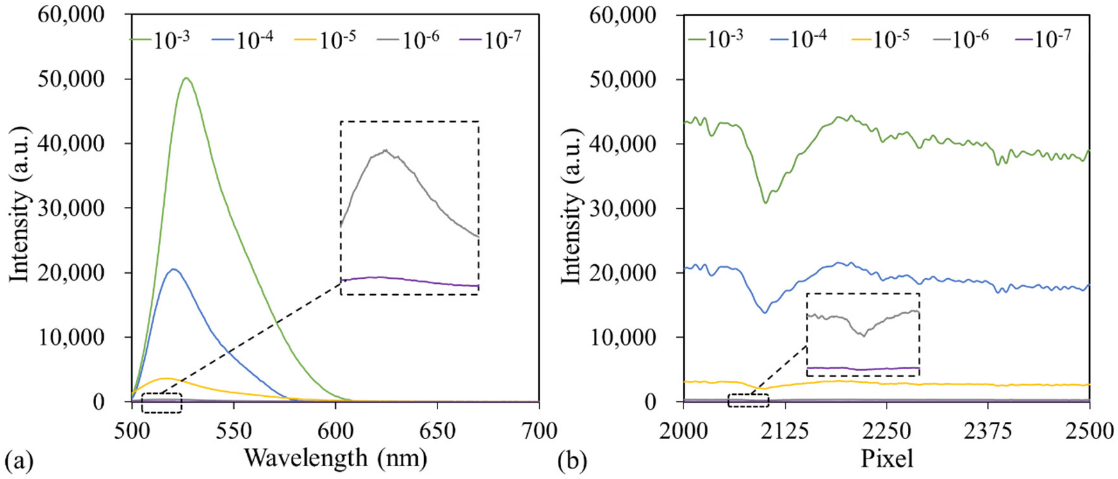

Figure S3. Fluorescence emission from different concentrations of FAM measured using (**a**) a spectrometer and (**b**) the proposed GGP-GMR/CCD system.

Experiments were performed three times to obtain a quantitative measurement. For each run, the intensities were normalized to that measured at the highest concentration. The dose response curves measured using the GGP-GMR/CCD system and spectrometer are displayed in Figures S4a and S4b, respectively. A comparison of the results (normalized intensity of the peak wavelength vs. the normalized intensity of the MICP) is illustrated in Figure S4c, where both the slope of the regression line and the coefficient of determination (R^2) approach 1, which indicates that the measurements obtained with the GGP-GMR/CCD system agree well with those obtained with a commercial spectrometer.

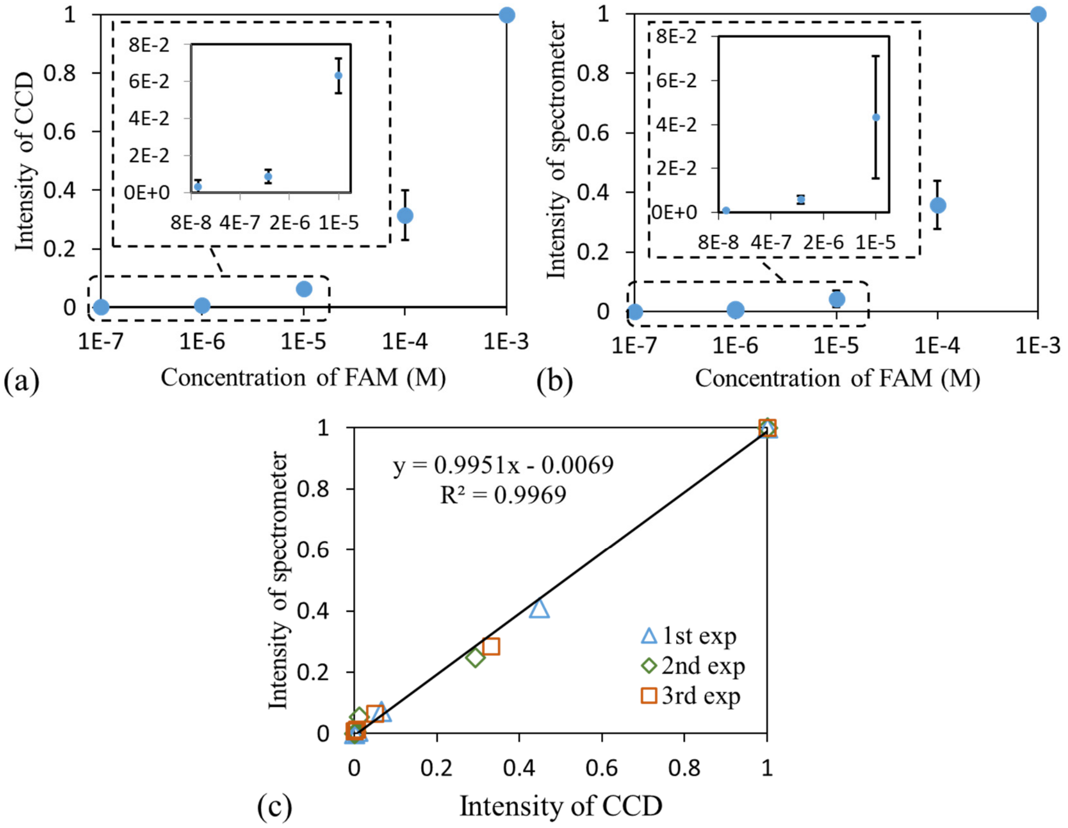

Figure S4. Dose response curve obtained using the (**a**) GGP-GMR/CCD system and (**b**) spectrometer. (**c**) This figure displays the correlation between the normalized intensity of the peak wavelength and that of the MICP.

In addition to the intensity, the peak wavelength and MICP shifted slightly with changes in the concentration, as displayed in Figures S3a and S3b. The peak wavelength shifted slightly from 536.76 to 527.02 nm when the concentration decreased from 10^{-3} to 10^{-7} M, as depicted in Figure S3a. The MICP also shifted from pixel number of 2115 to 2101 with decrease of concentration from 10^{-3} to 10^{-7} M. The variations in the peak wavelength and MICP with the concentration obtained from the three experimental runs are illustrated as blue and orange curves in Figure S5a, respectively. A comparison of the results between the measured MICPs and peak wavelength measured by the spectrometer is presented in Figure S5b, where the large value of the coefficient of determination (0.9995) indicates a strong correlation between the MICP and the peak wavelength. These fluorescence measurement results reassure that the location and intensity of the MICP can be used to correlate the peak wavelength and its intensity appropriately.

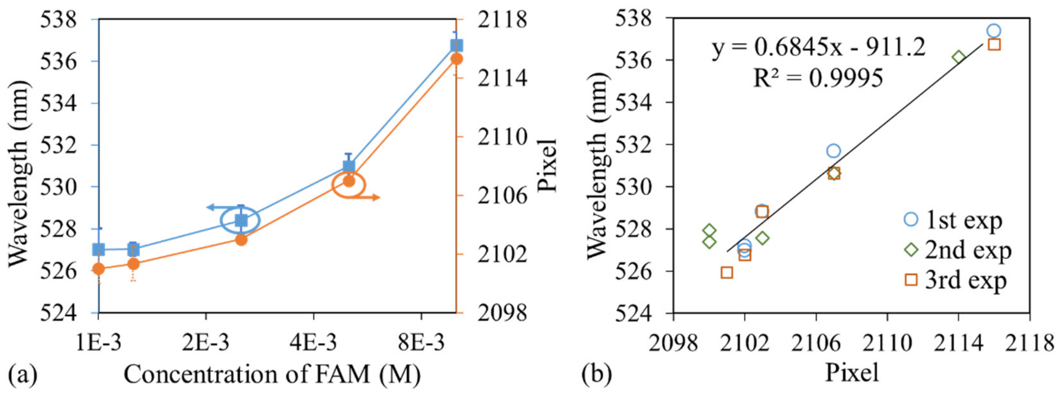

Figure S5. (**a**) Peak wavelength (blue) and measured MICP (orange) as a function of the concentration. (**b**) Correlation between the peak wavelength and the MICP.

Table S1. Composition of artificial urine.

Composition	Concentration (g/L)
Calcium chloride	0.44
Magnesium chloride hexahydrate	0.65
Sodium chloride	4.8
Sodium sulfate	2.3
Sodium citrate	0.65
Creatinine	1.1
Potassium dihydrogen phosphate	2.8
Ammonium chloride	1.0
Urea	25.0
